# Anaesthetic Consideration in Macroglossia Due to Lymphangioma of Tongue: A Case Report

**Published:** 2009-02

**Authors:** Anurag Tewari, Munish Munjal, Kamakshi, Shuchita Garg, Dinesh Sood, Sunil Katyal

**Affiliations:** 1 Assistant Professor, Department of Anaesthesiology & Resuscitation., Institute: Dayanand Medical College & Hospital, Ludhiana, Punjab, India. PIN: 141001; 2 Professor, ENT, Department of Anaesthesiology & Resuscitation., Institute: Dayanand Medical College & Hospital, Ludhiana, Punjab, India. PIN: 141001; 3 Senior Resident, Department of Anaesthesiology & Resuscitation., Institute: Dayanand Medical College & Hospital, Ludhiana, Punjab, India. PIN: 141001; 4 Intensivist, Critical Care, Department of Anaesthesiology & Resuscitation., Institute: Dayanand Medical College & Hospital, Ludhiana, Punjab, India. PIN: 141001; 5 Professor, Department of Anaesthesiology & Resuscitation., Institute: Dayanand Medical College & Hospital, Ludhiana, Punjab, India. PIN: 141001; 6 Professor&Head Department:Department of Anaesthesiology & Resuscitation., Institute: Dayanand Medical College & Hospital, Ludhiana, Punjab, India. PIN: 141001

**Keywords:** Airway management, Anaesthesia, Lymphangioma, Macroglossia

## Abstract

**Summary:**

Successful airway management of an infant or child with macroglossia prerequisites recognition of a potential airway problem. We describe our experience with a debilitated 13-year-old girl who presented with severe macroglossia, secondary to lymphangioma of the tongue. Along with the social discomfort she had inability to speak, eat or drink properly and exposure-induced dryness. Such patients are a challenge for the anaesthesiologists due to the anticipated difficult intubation associated with the oral mucosa occupying lesion. It also becomes pertinent to rule out any of the associated congenital anomalies. The importance of a thorough preoperative evaluation and attention to difficult intubation and maintenance of airway is emphasized. We endeavor to review the available literature regarding patient's perioperative management of such patients.

## Introduction

A child with markedly enlarged tongue presents a unique challenge to the anaesthesiologists. Techniques for managing difficult airway in children are different from those used in adults. The anaesthesiologist and otorhinolaryngologists team should plan and execute the perioperative airway management. We present a case of recurrent lymphangioma of tongue leading to macroglossia in a 13-year-old girl who had presented for hemiglossectomy.

The first accurate description of lymphangioma was given by Virchow in 1854. Lymphangiomas are benign hamartomatous tumors of the lymphatic channels. They present as developmental malformations arising from sequestration of lymphatic tissue that do not communicate with the rest of the lymphatic channels^1^. They can also occur in association with hemangioma.

Lymphangiomas have a marked predilection for the head and neck region, which accounts for about 75% of all cases and about 50% of these lesions are noted at birth and around 90% develop by 2 years of age^2^. They are known to be associated with Turner's syndrome, Noonan's syndrome, trisomies, cardiac anomalies, fetalhydrops, fetalalcohol syndrome, and Familial pterygium colli^2^.

Oral lymphangiomas may occur at various sites but they form most frequently on the anterior two-thirds of the tongue, which often result in macroglossia. It can also present in the palate, buccalmucosa, gingiva, and lip^3^,^4^.

## Case report

A 13-year-old female child presented to otorhinolayrngology department with enlargement of tongue for the last 1 year. It was insidious in onset. She gave a history of progressive difficulty in ingesting semi solid food. The girl had been operated for the same pathology, at the age of four months under general anaesthesia after oro-tracheal intubation. There were no records ofany perioperative complications then.

A thorough preoperative examination was done priorto surgery. The girl was poorly nourished, under weight (18 kg), anxious, embarrassed, apprehensive, and was unable to speak comprehensively. There was no history of respiratory difficulty, trauma, pain, bleeding or sudden increase in the size of lingual swelling. She could take only liquid diet. A good rapport was developed with her and she was explained in vernacular language the need for surgery and what she should expect in the operating room. Extensive examination of the other body systems revealed no relevant medical problems.

**Fig 1:**
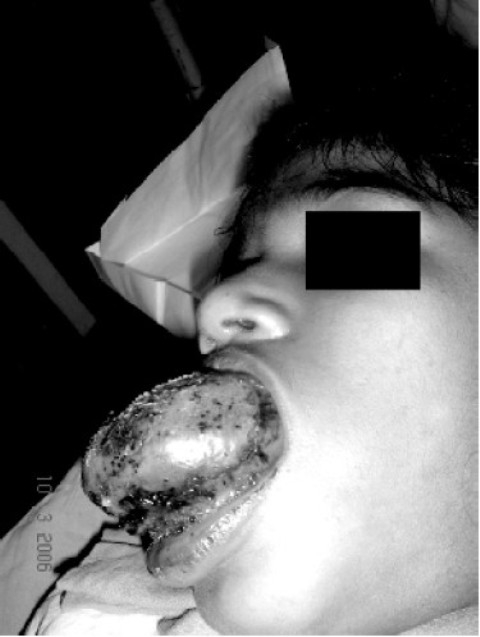
Lateral view of macroglossia in the patient

**Fig 2:**
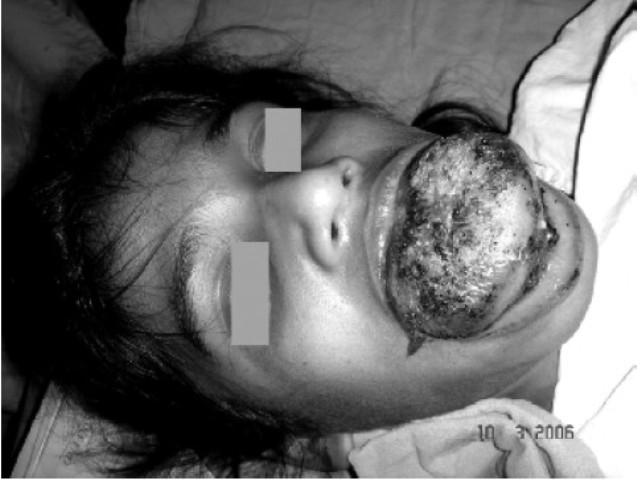
Anterio-posterior view of the macroglossia

Local examination revealed adiffusely enlarged tongue; protruding and keeping the mouth permanently open (Fig 1 & 2). Ulcerations over the dorsum of tongue on anterior part were present. The oro-dental hygiene was poor with the lower teeth completely compressed inside the swollen gums. Mouth opening, inter-incisor gap and Mallampatti grading could not be elicited due to the enlarged tongue. On palpation thetongue was tender and firm in consistency. All other congenital abnormalities were looked for and ruled out by the pediatrician. Preoperative blood analysis revealed a haemoglobin of 10mg/dL and a haematocrit of 30%. The serum electrolytes, ECG, and chest X-ray were normal. Fine needle aspiration cytology of the lingual swelling yielded only blood, hence a clinical diagnosis of hemangioma was made. The child was to be taken up for V-glossoplasty.

**Fig 3:**
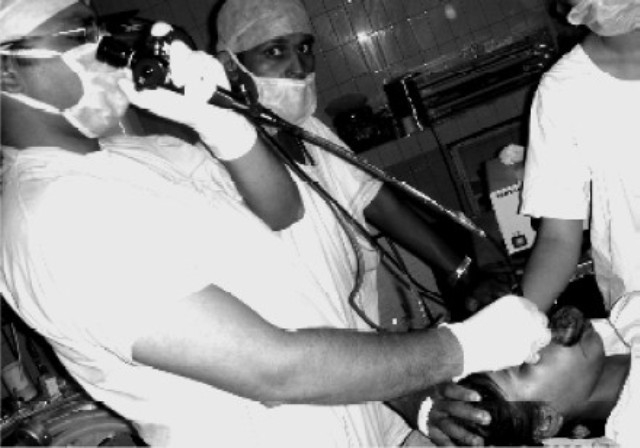
Awake fiberoptic intubation being carried out

**Fig 4:**
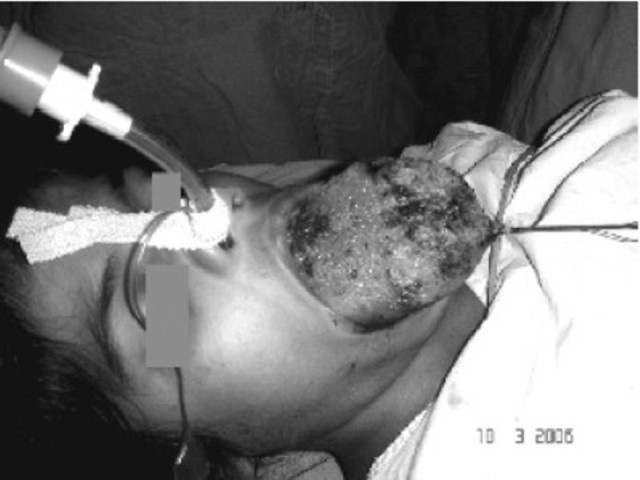
Endotracheal tube secured after proper confirmation of its placement

Premedication was given in the form of midazolam 1mg, glycopyrrolate 0.2mg and fentanyl 30μg intravenously prior to shifting her to operation room. All the preparation for anticipated difficult intubation/ventilation and tracheostomy were kept ready. Monitoring was initiated with continuous ECG, arterialoxygen saturation, temperature and non invasive blood pressure monitoring. The surgeons were asked to remain standby, in case if tracheostomy was required. Both the nostrils were instilled with xylometazoline drops. Preoxygenation was initiated using a large anatomical facemask (number 4). The focus was on “Awake intubation”, we did notgive any thing for induction… as that would have compromised the airway. The fibreoptic scope PentaxF1-10P2 was introduced through the right nostril (Fig 3). In the oral cavity it was difficult to manipulate the fiberscope due to the enlarged tongue. The neck of the patient had to be flexed for proper visualization of the glottis and then the cuffed endotracheal tube (ETT) number 6.5 was guided under vision into the trachea. Neuromuscular blockade was achieved with rocuronium and the ETT was secured after confirming bilateral airentry (Fig 4). She was maintained on propofol (10mg.kg^−1^.hr^−1^) and fentanyl infusion (10μg.hour^−1^) along with oxygen and nitrous oxide (ratio of 33:67).

The inverted Vshaped anterior half of the tongue was removed. The tumor had large cystic spaces filled with lymph like fluid. The part of tumor going into the base of the tongue was injected with sclerosing agent i.e. 25% dextrose. The tongue was then sutured in midline to give it normal shape. The surgery lasted for three hours and the total blood loss was 150ml. At the end of surgery the child was allowed to return back to spontaneous respiration and neuromuscular blockade was reversed after adequate return of muscle power and respiratory tidal volume. It was decided to keep her electively intubated and thereafter put on T-piece connected to the endotracheal tube with oxygen support 6L/minute. She was shifted to the post anaesthesia care unit where she was kept sedated with propofol and fentanyl. The trachea was extubated on the second postoperative day. She maintained her vitals and arterialoxygen saturation within normal levels without any support.

On histopathological examination a complex vascular malformation of the lymphangioma-hemangioma type, extensively involving the deep musculature of the tongue was found. There was extension into the base of tongue in background of variable muscle degeneration and marked fibrosis. The patient had no postoperative problems and was eventually discharged on the seventh day.

## Discussion

Lymphangiomas are hamartomatous, congenital malformations of the lymphatics. They are the result of sequestration of lymphatic tissue that has retained its potential for growth and do not communicate with other lymphatic tissue^5^. Lymphangioma can be classified into four categories: Lymphangioma simplex (lymphangioma circumscriptum): composed of small, thin-walled lymphatics. Cavernous lymphangioma: comprised of dilated lymphatic vessels with surrounding adventitia. Cystic lymphangioma (cystic hygroma):consisting of huge, macroscopic lymphatic spaces with surrounding fibrovascular tissues and smooth muscle. Benign lymphangioendothelioma (acquired progressive lymphangioma): lymphatic channels dissect through dense collagenic bundles^5^.

Occasionallesions demonstrate proliferation of lymphatic channels with another connective tissue component, primarily smooth muscle cells (lymphangiomyoma). It is derived embryologically from five primitive buds developing from the venous system which include paired jugular sacs, paired posterior sacs and a single retroperitoneal sac^5^. Cervical lesions in a child can cause dysphagia and airway obstruction which is rare in adults^6^. In the present case, the swelling was noticed since birth for which she had undergone palliative surgery at the age of four months. There was resurgence of the lingual swelling in the last one year.

The anterior two-thirds on the dorsal surface of tongue is the most common site for intra-oral lymphan-giomas leading to macroglossia^3^,^4^. These patientst end to have speech disturbances, poor oral hygiene, and bleeding from tongue associated with oral trauma^7^. In our case, macroglossia resulted in lesions on the dorsal surface of tongue, improper phonation and poor oral hygiene.

The various treatment modalities for lymphan-gioma are surgical excision, radiation therapy, cryotherapy, electrocautery, sclerotherapy, steroid administration, embolization and ligation^6^,^8^, laser surgery with Nd-YAG^9^–^11^, CO_2_^12^,^13^, and radio-frequency tissue ablation technique^14^. Surgical excision is the preferred treatment for cystic hygroma but complete removal is not possible because of the multiple finger like projec-tions in the surrounding tissues^15^,^16^.

Since lymphangioma is primarily a disease of childhood, the paediatric dentist mightbe the first healthcare professional to encounter this lesion. An early diagnosis and intervention would help in reducing functional and psychological disturbances and also cosmetic disfigurement. A complete and frank discussion with the parents (and child if appropriate) should always include the anaesthetic and surgical protocol along with the associated risks. It is pertinent that the possibility of tracheostomy and, indeed, of failure to secure the airway should be mentioned.

Awake intubation may not be easily performed in children since cooperation is quintessential. Premedication and preoxygenation should be followed by inhalation of either halothane or sevoflurane, in a spontaneously breathing patient. Muscle relaxants should be withheld until theair way is secured. Intubation should be performed under deep inhalational anaesthesia. Use of a muscle relaxant during induction of anaesthesia may result in a situation where we may land up in either a difficult to ventilate and difficult to intubate scenario, and may therefore warrant securing of a surgical airway rapidly. Hence maintenance of spontaneous breathing allows a way out, should there be a problem in securing the airway^17^,^18^.

Visualization of the larynx is better in deeper planes of anaesthesia. If difficult for whatever reasons, the anaesthesiologist must have a secondary plan of how to proceed. If the surgical requirement is not pressing, one must consider postponing the procedure, but if the procedure is essential then alternative means must be available to accomplish endotracheal intubation. In these groups of patients, certain maneuvers using conventional equipment do not always succeed, hence fibreoptic intubation techniques are often necessary^18^.

Adult fibreoptic bronchoscopes have an outer diameter of around 3.5-4.0 mm and thus can take realistically a size 4.0-4.5 endotracheal tube loaded onto them. Ultrathin fibrescopes have an outer diameter of 2.2 mm so a 2.5 mm endotracheal tube can be rail-roaded over them^18^. The optical quality of these scopes is good but it has no suction channel and secretions have to be aspirated with a suction catheter^18^.

Anaesthesia can be maintained via a nasal airway or via a specially adapted facemask. The bronchoscope can then be inserted into the mouth and the larynx visualized. The laryngeal mask airway can also be used. Use of this device in anaesthetic practice can avoid the need for intubation^19^, but should intubation be deemed necessary it provides a superb airway conduit. The laryngeal mask airway in paediatric patients with difficult airway is an excellent aid to visualize the larynx and endotracheal intubation^20^.

Blind techniques are possible with either a gum elastic bougieor an endotracheal tube^21^. Fibreoptic techniques depend on adaptation either of the laryngeal mask airway (split^22^ or shortened^23^) or of the mode of endotracheal passage (telescoping the tube over the fiberscope^24^ or a wire technique^25^). Shortening the laryngeal mask airway and splitting are other methods to the same end, allows the anaesthesiologist to advance the endotracheal tube through the laryngeal mask airway which can then be removed without hazarding the tube.

The guidewire technique allows the anaesthesiologist to insert a conventional adult fibreoptic bronchoscope to gaina view of the larynx and use the suction facility. The suction channel can then be employedto facilitate passage of along guide wire into the trachea. This avoids the need to preload an endotracheal tube onto the fibrescope and railroad the tube into thetrachea through the laryngeal mask airway, a difficult procedure, and even then the laryngealmask airway must be removed to allow proper fixation of the tube.

When the patient is breathing deeply, spontaneously, on sevoflurane or halothane, the fibreoptic bronchoscope is introduced and a view of the cords is obtained. Lidocaine 2-3 mg.kg^−1^ may be sprayed via the suction channel of the fibreoptic scope onto the cords. The fibreoptic scope is manipulated through the cords into the trachea until the bifurcation of the trachea is visible. Fibreoptic intubation should not be undertaken lightly in children.
